# Multiglandular Parathyroid Disease

**DOI:** 10.3390/life12081286

**Published:** 2022-08-22

**Authors:** Grzegorz Kowalski, Grzegorz Buła, Adam Bednarczyk, Agata Gawrychowska, Jacek Gawrychowski

**Affiliations:** Department of General and Endocrine Surgery, School of Medicine with the Division of Dentistry in Zabrze, Medical University of Silesia, 40-055 Katowice, Poland

**Keywords:** multiglandular parathyroid disease, sporadic primary hyperparathyroidism, parathyroidectomy

## Abstract

Introduction: Multiglandular parathyroid disease (MGD) is an uncommon cause of primary hyperparathyroidism (pHPT) and has been reported in the literature in 8–33% of patients with pHPT. The aim of our study was to review the clinical characteristics and management of MGD and evaluation of surgical treatment failures. Methods: We performed a retrospective study of 163 patients with pHPT undergoing parathyroidectomy (PTX) at the Department of General and Endocrine Surgery between 1983 and 2018. All these patients were diagnosed with MGD. This group of patients was compared with a group of 856 patients with solitary disease operated for pHPT in the same period. Results: Among 163 patients—127 (79%) of them had two lesions, 28 (16%) had three, and 8 (5%) four. They were prevalently women over the age of 50. The diagnosis was based on PTH and ionized calcium studies and used sestamibi technetium-99m scintigraphy (MIBI) as well for us. Treatment was surgical. Conclusions: Parathyroidectomy (PTX) for multiglandular parathyroid disease (MGD) is associated with a higher operative risk of failure compared to solitary disease. Preoperative diagnosis and localization of the parathyroid glands is an extremely important element of treatment. Diagnosis is based on PTH and calcium levels. Ultrasonography (USG), MRI, and scintigraphy are very helpful in diagnosis. Mediastinal multiglandular parathyroid disease (MGD) is associated with increased surgical treatment failures. The treatment is surgical and consists of the removal of the masses or complete parathyroidectomy. Based on this study, we support the existence of multiple adenomas and advocate the removal of only macroscopically enlarged parathyroid glands in patients with primary hyperparathyroidism.

## 1. Introduction

Multiglandular parathyroid disease (MGD) is usually caused by benign parathyroid tumors that lead to hyperparathyroidism. According to literature, in 8–33% of patients with primary hyperparathyroidism (pHPT) have multiglandular disease [1,2]. Clinical presentation of patients with MGD is usually due to increased values of ionized calcium and is very similar to patients with single parathyroid lesions [3,4,5]. As a result, the clinical picture and presenting symptoms alone is not a base for the diagnosis of MGD [6,7]. Most cases involved a single abnormal parathyroid gland located in a usual neck site, but many such changes occur in other sites including the thymus gland, the retroesophageal space, or the thyroid gland. Sometimes, parathyroid lesions have been reported in the pericardium or soft and adipose tissues of the mediastinum up to the angle of the jaw [8,9]. The localization of such revealed lesions is varying. Preoperatively imaging of choice are usually scintigraphy, high-resolution ultrasonography, CT scanning, and MRI [10,11,12]. Usually, the first line test is sestamibi technetium-99m scintigraphy (MIBI), and later, well-performed USG can be a confirmatory method [13,14]. Moreover, the use of the single-photon emission computed tomography (SPECT) technique and 4D-CT provides the high possibility of anatomic detail and affords the high likelihood of achieving a safe and successful operation [15,16]. MRI are useful in the detection of particularly ectopic extra parathyroid mediastinal lesions [5,17]. The treatment of MGD is surgery-parathyroidectomy (PTX) with the use of an intraoperative PTH (iPTH) level [18,19,20]. The aim of our study was to review the clinical characteristics and management of MGD and the evaluation of surgical treatment failures.

## 2. Materials and Methods

This is a retrospective study of 163 patients—106 (65%) women and 57 (35%) men aged from 21 to 76 years (mean 55 years), operated between 1983 and 2018 on primary hyperparathyroidism (pHPT) caused by multiglandular parathyroid disease (MGD). All patients prior to the surgery underwent diagnostic protocol to confirm primary pHPT. Patients were identified based on operation protocols and histopathological findings regarding the number of lesions, their localization, and biochemical and surgical results. Parathyroid localization studies included up until 2004 the ultrasound and subtraction technique; from 2005, sestamibi technetium-99m scintigraphy (MIBI) was used, and from 2010, the single-photon emission computed tomography (SPECT) technique was usually been utilized. Two positive imaging studies were always required and performed for the estimation of lesion localization. The surgical policy of parathyroidectomy in our institute was focused on routine four glands exploration. Of all successfully operated patients, normocalcemia (ionized calcium level 0.98–1.13 mmol/L) lasting was persistent in a span of 1 to 26 years (mean 6.2). In 48 (29%) patients, the multiple lesions were synchronous, hyperplasia/cancer or hyperplasia/adenoma. In total, 148 (90%) patients underwent removal of all the enlarged parathyroid glands in one operation, and 13 (8%) patients had removed one lesion during the first operation—reoperation for persistent hypercalcemia was performed, and the remaining parathyroid lesions ware resected. Two patients (1%) developed recurrent hyperparathyroidism and required reoperations. After surgical treatment, all patients remained closely monitored for any side effects or therapy failure. The patients were seen at least four times a year—i.e., every three months within the first two years and every six months thereafter. Physical and biochemical examinations were also performed. The levels of ionized calcium and PTH were measured and, if hypercalcemia was persisting after surgery, patients were closely monitored. We arbitrarily divided cases of hypercalcemia after prior surgery into persistent (defined as hypercalcemia recurring within six months of the initial operation) or recurrent (hypercalcemia recurring after six months of normocalcemia). 

### Methods of Statistical Analysis

All the collected data was included in a spreadsheet in Microsoft Office Excel 2019. The statistical analysis was performed in Statistica 12.5. After establishing and classification of the data using a Kolmogorov–Smirnov test, we used Student’s t-test to compare data with normal distribution, and for the data of non-normal distribution, we used the Mann–Whitney U test. The results are presented as percentage changes, full numbers, means, and standard deviations.

## 3. Results

In this clinical study we investigated a cohort of patients with multiglandular parathyroid disease (MGD) during primary hyperparathyroidism (pHPT) surgery. Our findings demonstrate that MGD was diagnosed in 163 (16%) patients with pHPT. We evaluated operated-on patients for parathyroid MGD and carried out comparisons with a group of 856 patients with single parathyroid lesions. Multiple lesions were predominantly found in women, with a prevalence of 65% (men 35%). In the group of patients with diagnosed multiple lesions with characteristics of hyperplasia, women represented 71% of cases. Most patients were diagnosed after the age of 50 years. Multiple lesions were mostly diagnosed in women after 50 years old (52%). In males, lesions were also predominantly diagnosed after 50 (55%). We discovered that in the group of patients with MGD, the most represented within the entire group was the inferior right parathyroid gland, with 53 cases. The inferior left parathyroid gland was operated in 45 cases, superior left in 32 cases, and superior right in 33 cases—implying that multiple parathyroid lesions are usually found in the right part of the neck when parathyroid glands are not ectopic. One hundred sixty-one (99%) patients with normal or ectopic localizations of multiple parathyroid lesions ware symptomatic. Fatigue, muscle or bone weakness, and loss of appetite were the most-observed symptoms. No symptoms were recorded among the remaining two (1%) patients (*p* < 0.05) (Table 1).

After final diagnosis, all patients underwent surgical treatment. Treatment consisted of removing all parathyroid lesions or, if it was necessary, total parathyroidectomy. MGD was diagnosed with the highest occurrence of double lesions. Overall, double lesions occurred in 127 (78%), triple lesions in 28 (17%), and four lesions in 8 (5%). In two cases (1%), we discovered multiple parathyroid lesions with cancer cells. Most of the multiple lesions were in the neck 146 (90%), and others were in the mediastinum 17 (10%). The mediastinal localization concerned patients with double—13 (8%)—and triple—4 (2.5%)—lesions of the parathyroid glands (Table 2). 

According to histopathological evaluation of the lesions, we can distinguish multiple lesions with characteristics of hyperplasia and adenomas, with the presence of carcinoma. Histopathological findings imply that hyperplasia and adenoma are the most common types of multiple lesions of the parathyroid glands. In 54 (33%) of patients from this group, we found multiple lesions with adenomas—of which 44 (81%) had double lesions, 7 (13%) had triple lesions and 3 (6%) had four lesions. In 155 (95%) of patients from this group, we found multiple lesions with characteristics of hyperplasia—of which 119 (77%) had double lesions, 28 (18%) had triple lesions, and 8 (5%) had four lesions (Table 3). 

The analysis of biochemical examination results indicated elevated serum ionized calcium and parathyroid hormone (PTH) levels within the entire group prior to operation without distinction of etiologies, number of lesions, or localizations. Most of the patients had severe hyperparathyroidism (blood ionized calcium level > 1.13 mmol/L and PTH level > 60 pg/mL) and symptoms of hypercalcemia. The levels of PTH were in 29% below 100, in 20% between 150–200, and in 26% above 200 pg/mL. Ionized calcium levels were mostly between 1.5–2.0 mmol/L prior to the treatment (82%) (Table 4). 

After a parathyroidectomy was performed, both PTH and calcium levels decreased. Calcium levels had normalized postoperatively and remained within the normal range for 12 months or longer. In patients where the levels stayed elevated, further diagnostic methods to diagnose the cause were undertaken. A total of 15 (9%) patients required remedial surgery due to persistent or recurrent hypercalcemia. There were four (2%) cases of persistent hypercalcemia caused by lesions of the neck and nine (6%) cases in the mediastinum. Recurrent hypercalcemia was found in two (1%) patients due to a gland remaining within the mediastinum. Comparing patients with solitary disease and multiple parathyroid lesions, we found a significantly higher incidence of persistent and recurrent hypercalcemia among patients with MGD (*p* < 0.001) (Table 5). In addition, in patients with multiple parathyroid lesions, we found a higher incidence of surgical treatment failures, and more often, the reoperation was necessary in the mediastinal location (*p* < 0.001).

Finally, out of the group of 163 patients with MGD, no failures in the surgical treatment of pHPT were reported. Until now, all patients with multiple parathyroid lesions have normal levels of PTH and calcium and no symptoms of hypercalcemia.

## 4. Discussion

Multiglandular parathyroid disease (MGD) is one of the causes of primary hyperparathyroidism (pHPT). The lesions are quite hard to diagnose and distinguish from solitary lesions or carcinoma. Some studies show that majority of MGD consist of double lesions, and only sporadically, bigger numbers are observed [1,2]. The multiple parathyroid gland disease is defined by Harness and others as: “More than one and fewer than 4 enlarged parathyroid glands at operation, operative finding of at least one normal parathyroid gland, evidence of neither MEN or familial hyperparathyroidism and permanent normocalcemia after resection of enlarged parathyroid glands” [3,13]. The most common genetic mutation responsible for formation of sporadic adenomas is cyclin D1/PRAD1 gene mutation and was found in 20–40% of patients [5]. Additionally, some studies suggest that previously performed head and neck radiation procedures can be a predisposing factor for the development of adenomas [6]. Some genetic predispositions were observed to coexist with the formation of adenomas in small groups of patients—most commonly, multiple endocrine neoplasia syndrome type 1, more rarely, multiple endocrine neoplasia type 2, and sporadically the hyperparathyroidism-jaw tumor syndrome. Those can predispose to the formation of multiple adenomas on the hyperplastic parathyroid gland [7].

Parathyroid lesions are more common in females (three times more often than in men) 50–70 years old [4,11]. Additionally, some studies suggest that a previously performed head and neck radiation procedure can be a predisposing factor for the development of adenomas [4,6,14]. The localization of multiple parathyroid adenomas is varying. They can occur in both superior and inferior parathyroid glands on the left or right side [1,8]. Single adenomas are monoclonal lesions arising from a single precursor, and multiple in the majority are polyclonal [10]. From a histological point of view, adenomas are mostly composed of chief cells, but we can also find oxyphil cells, oncocytes, transitional oncocytes, or a mixture of these cell types [11]. Additionally, the distinction of multiple adenomas from hyperplasia is quite difficult. In hyperplasia, all four glands are changed and according to definition multiple adenomas affect less than four glands, but more than one. 

There is conflicting evidence and statements that draw the question if multiple adenomas are not a separate entity but just asymmetrical hyperplasia, according to some scientists [9]. The clinical presentation of patients with multiple adenomas are usually due to increased values of calcium and are very similar to patients with single parathyroid adenomas. The clinical picture and presenting symptoms alone are not a base for the diagnosis of multiple adenomas as studies suggest [12]. The most common symptoms of primary hyperparathyroidism that patients observed were bone pain, fatigue, anorexia and weight loss, abdominal pain, and depression. Helpful for diagnosis can be blood studies, measurements of PTH, and calcium that is usually elevated. Unfortunately, according to our study, these values cannot be the only diagnostic method to diagnose multiple lesions. PTH levels were very variable ranging from <100 until even more than 500 pg/mL. Calcium levels were predominantly within 1.5–2.0 mmol/L. Laboratory studies show elevated levels of calcium, PTH, and usually decreased levels of phosphate. Unfortunately, the results of clinical tests of patients with multiple adenomas are quite similar to those with a single adenoma or hyperplasia and cannot be considered as a valuable diagnostic factor [13]. The diagnosis of MGD preoperatively is quite difficult. Firstly, the laboratory studies are performed to measure calcium, PTH, and phosphorus levels to confirm diagnosis of primary hyperparathyroidism. Palpation of the neck can, in some cases, reveal enlarged masses, but it is not a predictive factor for multiple adenomas, although it should raise the suspicion of carcinoma [13,14]. The preoperative diagnosis of MGD is very important to reduce the risks of surgery failure. The best method is sestamibi technetium-99m scintigraphy (MIBI) and single-photon emission computed tomography (SPECT) technique or 4D-CT for the localization of the mass. The USG of the parathyroid can show presence of mass and can be a good preoperative diagnostic method. MRIs are useful in the detection of extra parathyroid lesions—particularly ectopic mediastinal lesions [7,9,11]. Nowadays, high detection rates can be obtained with 18F-fluorocholine (or 11C-choline) PET/CT. These PET tracers open new avenues for radionuclide imaging of parathyroid glands. Equally important is the genetic study that is now easily carried out with the NGS technique on several genes [5,19]. To distinguish adenoma from carcinoma, biopsy in parathyroid carcinoma can lead to tumor dissemination. It is important to check all parathyroids for the presence of masses so the therapy can be planned based on all the information we can obtain. Even with the use of all available modalities, it is quite hard to predict the presence of multiple parathyroid lesions in some cases [10,12]. Some authors proposed scoring systems for the prediction of multiple parathyroid lesions’ existence. Kebebew et al., at San Francisco proposed a dichotomous scoring model that took into consideration preoperative calcium levels (>3 mmol/L), parathyroid hormone levels (>2 times higher than normal), positive USG, and sestamibi scans [7]. According to the scoring, patients with scores of three or higher almost always have single adenoma and can be treated with parathyroidectomy (PTX) without additional intraoperative studies. Patients with a score of less than three should undergo additional studies to confirm or exclude the presence of multiple lesions [17,18]. The therapy is based on a surgical approach. In MGD, complete removal of all enlarged parathyroid glands is necessary [15,19]. Appropriate surgical therapy of MGD should consist of a bilateral approach in most patients. Unilateral neck exploration guided by preoperative imaging should be reserved for selected patients, performed by an experienced endocrine surgeon, and monitored by intraoperative parathormone assay. 

Surgery in MGD poses several challenges. The diagnosis is often not clear, and preoperative localization is helpful. Hyperplastic glands are typically much smaller than solitary parathyroid adenomas—therefore, they are more difficult to identify. It can be difficult to distinguish a small hyperplastic gland from a normal parathyroid. Treatment in MGD requires more surgical experience and judgment compared to the excision of a solitary adenoma [5,9]. Recurrent/persistence pHPT occurs more frequently in patients with double adenomas; hence, in situations where a double adenoma has been identified, the surgeon should have a high index of suspicion during surgery and postoperatively for the possibility of a four-gland disease [1,5,19]. All tissues that were removed should be sent for pathology for further evaluation and the exclusion of the presence of cancerous cells. If cancer is confirmed by a pathologist, the treatment should become oncological, because early diagnosed and treated parathyroid carcinoma can be cured. After therapy, patients should be monitored for PTH and calcium levels for at least a year and checked for the presence of any hypercalcemia symptoms. Long-term follow-up is mandatory in a patient with MGD. After surgical therapy, the levels of PTH and calcium should be checked, and patients should be informed about the symptoms of hypercalcemia so they are aware of its occurrence [20].

## 5. Conclusions

1. Parathyroidectomy (PTX) for multiglandular parathyroid disease (MGD) is associated with a higher operative risk of failure compared to solitary disease.

2. The preoperative diagnosis and localization of the parathyroid glands is an extremely important element of treatment.

3. Mediastinal multiglandular parathyroid disease (MGD) is associated with increased surgical treatment failures. 

## Figures and Tables

**Table 1:** Patients’ characteristics operated for single and multiple parathyroid lesions.

Factor			Patients/Abnormalities		*p*-Value
			Single n = 856	Multiple n = 163	
Sex	M		208	57	0.38
	F		648	106	
Age (years)	M		21–79 (55.0)	24–76 (55.8)	0.46
	F		19–81 (55.0)	21–71 (54.2)	0.39
Symptoms	Yes		760	161	0.004
	No		96	2	
Localization	left	superior	140	32	0.013
		inferior	258	45	
	right	superior	136	33	0.013
		inferior	322	53	

**Table 2:** Patients’ characteristics operated for multiple parathyroid lesions.

Factor		Localization/No. of Patients		*p*-Value
		Neck	Mediastinum	
		n = 146	n = 17	
Lesion	benign	145	16	0.104
	malignant	1	1	
No. of lesions	2	114	13	0.070
	3	24	4	
	4	8	0	

**Table 3:** Histopathological findings patients operated for multiple parathyroid lesions.

Histopathological Finding	Patients/Abnormalities			*p*-Value
	All n = 163	Neck n = 146	Mediastinum n = 17	
2× hyperplasia + cancer	1	1	0	0.792
1× hyperplasia + cancer	1	0	1	<0.001
2× hyperplasia	82	73	9	0.084
2× adenoma	8	5	3	<0.001
3× hyperplasia	20	18	2	0.518
hyperplasia + adenoma	36	34	2	0.819
4× hyperplasia	5	5	0	0.560
2× hyperplasia + adenoma	6	6	0	0.520
3× hyperplasia + adenoma	3	3	0	0.648
2× adenoma + hyperplasia	1	1	0	0.792

**Table 4:** Levels of ionized calcium and PTH in blood serum of patients undergoing surgical treatment for single and multiple parathyroid lesions.

Number of Affected Parathyroid Glands	Examination	Before Operation pg/mL–mmol/L		24 h after Operation pg/mL–mmol/L		*p*-Value
		Value Levels	Average	Value Levels	Average	
Single	PTH	45.6–1865	168.51	2–176.8	22.74	<0.001
	Calcium	1.3–2.98	1.78	0.78–2.01	1.13	<0.001
Multiple						
2× lesions	PTH	44.6–540	140.54	1.89–159.1	20.1	<0.001
	Calcium	1.1–2.68	1.68	0.76–1.77	1.12	<0.001
3× lesions	PTH	45.6–665	158.11	2.6–166.8	42.74	<0.001
	Calcium	1.3–2.88	1.78	0.87–2.11	1.13	<0.001
4× lesions	PTH	54.2–865	282.07	2.8–176.9	46.52	<0.001
	Calcium	1.4–2.89	1.79	0.89–2.21	1.24	<0.001

**Table 5:** Persistent and recurrent hypercalcemia patients with single and multiple parathyroid lesions.

Lesions	Localization	*p*-Value	Hypercalcemia		*p*-Value
			Persistent	Recurrent	
Single n = 856	Neck n = 6	<0.001	2 (0.2%)	4 (0.5%)	0.143
	Mediastinum n = 10		7 (0.8%)	3 (0.4%)	
Multiple n = 163	Neck n = 4		4 (2.5%)	0	<0.001
	Mediastinum n = 11		9 (5.5%)	2 (1.2%)	

## Abbreviations

MGD	multiglandular parathyroid disease
pHPT	primary hyperparathyroidism
PTX	parathyroidectomy
MIBI	sestamibi technetium-99m scintigraphy
USG	ultrasonography
PTH	parathormone
SPECT	single-photon emission computed tomography
iPTH	intraoperative PTH
